# Tissue mRNA for S100A4, S100A6, S100A8, S100A9, S100A11 and S100P Proteins in Colorectal Neoplasia: A Pilot Study

**DOI:** 10.3390/molecules26020402

**Published:** 2021-01-14

**Authors:** Eva Peterova, Jan Bures, Paula Moravkova, Darina Kohoutova

**Affiliations:** 1 2nd Department of Internal Medicine–Gastroenterology, Charles University, Faculty of Medicine in Hradec Kralove, University Hospital, Sokolska 581, 500 05 Hradec Kralove, Czech Republic; PETEROVE@lfhk.cuni.cz (E.P.); paula.moravkova@fnhk.cz (P.M.); KohoutovaD@lfhk.cuni.cz (D.K.); 2 Department of Medical Biochemistry, Charles University, Faculty of Medicine in Hradec Kralove, Simkova 870, 500 01 Hradec Kralove, Czech Republic; 3 The Royal Marsden Hospital NHS Foundation Trust, Fulham Road, Chelsea, London SW3 6JJ, UK

**Keywords:** colorectal neoplasia, large intestine, mRNA, S100 proteins

## Abstract

S100 proteins are involved in the pathogenesis of sporadic colorectal carcinoma through different mechanisms. The aim of our study was to assess tissue mRNA encoding S100 proteins in patients with non-advanced and advanced colorectal adenoma. Mucosal biopsies were taken from the caecum, transverse colon and rectum during diagnostic and/or therapeutic colonoscopy. Another biopsy was obtained from adenomatous tissue in the advanced adenoma group. The tissue mRNA for each S100 protein (S100A4, S100A6, S100A8, S100A9, S100A11 and S100P) was investigated. Eighteen biopsies were obtained from the healthy mucosa in controls and the non-advanced adenoma group (six individuals in each group) and thirty biopsies in the advanced adenoma group (ten patients). Nine biopsies were obtained from advanced adenoma tissue (9/10 patients). Significant differences in mRNA investigated in the healthy mucosa were identified between (1) controls and the advanced adenoma group for S100A6 (*p* = 0.012), (2) controls and the non-advanced adenoma group for S100A8 (*p* = 0.033) and (3) controls and the advanced adenoma group for S100A11 (*p* = 0.005). In the advanced adenoma group, differences between the healthy mucosa and adenomatous tissue were found in S100A6 (*p* = 0.002), S100A8 (*p* = 0.002), S100A9 (*p* = 0.021) and S100A11 (*p* = 0.029). Abnormal mRNA expression for different S100 proteins was identified in the pathological adenomatous tissue as well as in the morphologically normal large intestinal mucosa.

## 1. Introduction

The etiology of sporadic colorectal cancer is multifactorial, and its pathogenesis is complex, not yet fully understood in detail [1,2,3,4,5]. Colorectal carcinogenesis is a multistep process in which calcium ions and calcium signaling play an important role in the control of the proliferation and differentiation of epithelial cells [6,7]. 

S100 proteins are small calcium-binding proteins that function as cell-type-specific mediators of Ca^2+^ signaling. S100 proteins are involved in a number of processes inside the cell but also at the extracellular level. Their participation in specific steps toward carcinogenesis is well-known. S100 proteins are involved in colorectal carcinogenesis through different mechanisms; they enable the proliferation, invasion and migration of tumor cells and affect apoptosis. S100 proteins also increase angiogenesis and activate the NF-κβ signaling pathway [8,9,10]. 

In our previous study, we investigated the serum concentrations of different S100 proteins in colorectal neoplasia. Patients with colorectal neoplasia had significantly lower serum concentrations of S100A6 and S100A11 and significantly higher serum concentrations of S100A8 compared to controls [11]. Overexpression of mRNA encoding S100 proteins synthesis was investigated in both colorectal cancer cell lines and surgically resected specimens of colorectal cancer in several studies [12,13,14,15,16]. 

The aim of our present study was to evaluate tissue mRNA encoding S100 proteins in patients with non-advanced and advanced colorectal adenoma in both, healthy intestinal mucosa and tissue of the colorectal neoplasia.

## 2. Results

In total, 18 biopsies were obtained from controls (6 individuals; caecum, transverse colon, rectum), 18 from patients with non-advanced adenoma (6 individuals) and 30 from patients with advanced adenoma (10 individuals). In nine patients with advanced adenoma, a biopsy sample was also taken from advanced colorectal neoplasia tissue. The mRNA for S100A4, S100A6, S100A8, S100A9, S100A11 and S100P was investigated.

**Figure 1:**
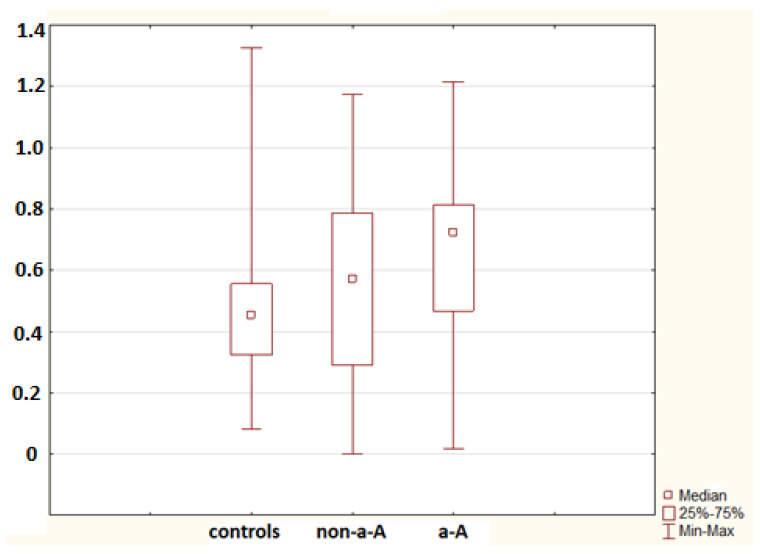
mRNA for S100A6 in controls (18 samples), the non-advanced adenoma group (non-a-A; 18 samples) and the advanced adenoma group (a-A; 30 samples). A statistically significant difference was found between controls and a-A (*p* = 0.012).

No difference in the mRNA for S100A4 was found between controls and the non-advanced adenoma group and between controls and the advanced adenoma group (*p* = 0.886 and *p* = 0.133, respectively). A significant difference in the mRNA for S100A6 was confirmed between controls and the advanced adenoma group (*p* = 0.012); no difference was found between controls and the non-advanced adenoma group (*p* = 0.351; Figure 1). A statistically significant difference in the mRNA for S100A8 was identified between controls and the non-advanced adenoma group (*p* = 0.033); no difference was confirmed between controls and the advanced adenoma group (*p* = 0.246).

**Figure 2:**
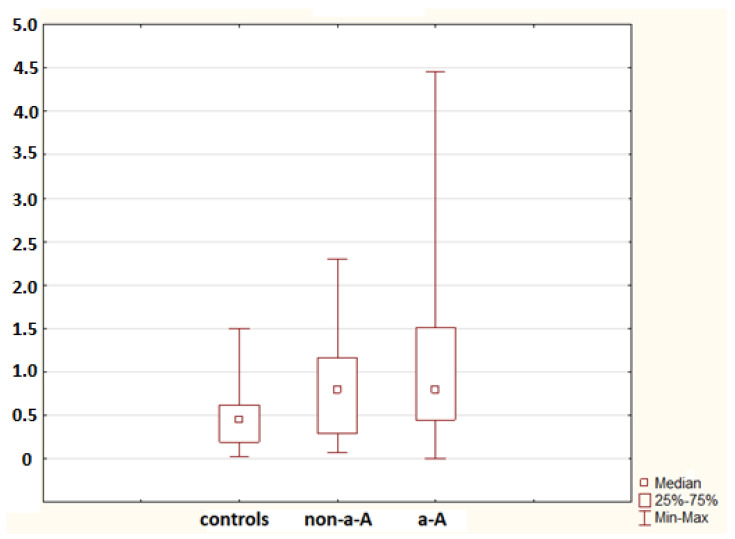
The mRNA for S100A11 in controls (18 samples), the non-advanced adenoma group (non-a-A; 18 samples) and the advanced adenoma group (a-A; 30 samples). A statistically significant difference was found between controls and a-A (*p* = 0.005).

**Figure 3:**
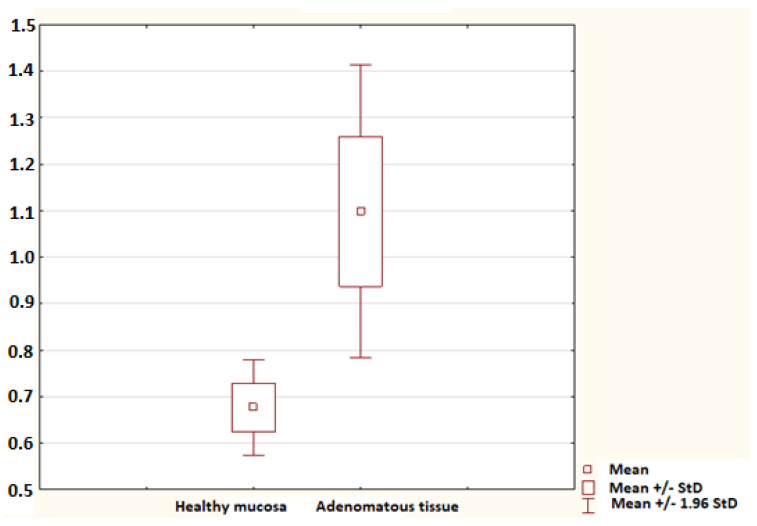
The mRNA for S100A6 in the healthy mucosa of the advanced adenoma group (healthy mucosa; 30 samples) and advanced adenomatous tissue (adenomatous tissue; 9 samples). A statistically significant difference was found (*p* = 0.002).

**Figure 4:**
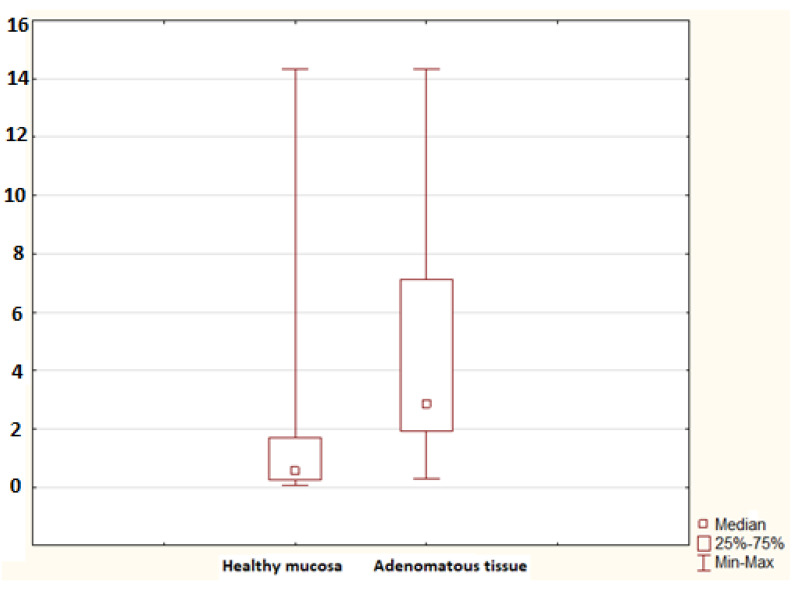
The mRNA for S100A8 in the healthy mucosa of the advanced adenoma group (healthy mucosa; 30 samples) and advanced adenomatous tissue (adenomatous tissue; 9 samples). A statistically significant difference was found (*p* = 0.002).

**Figure 5:**
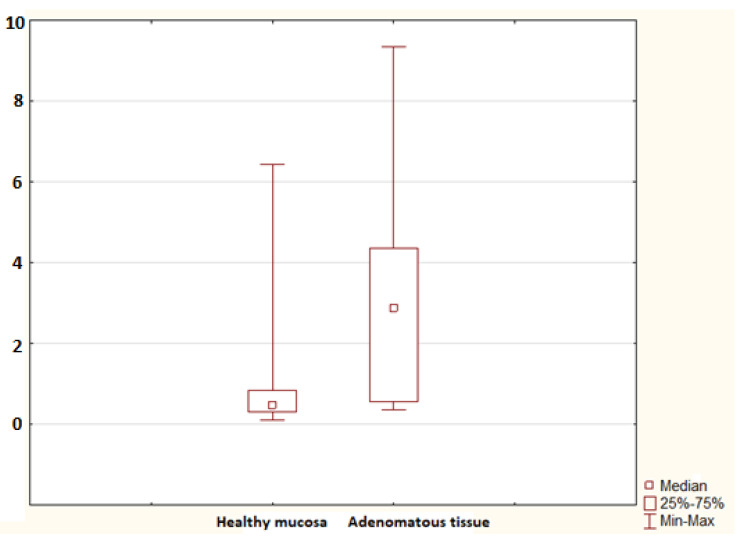
The mRNA for S100A9 in the healthy mucosa of the advanced adenoma group (healthy mucosa; 30 samples) and advanced adenomatous tissue (adenomatous tissue; 9 samples). A statistically significant difference was found (*p* = 0.021).

**Figure 6:**
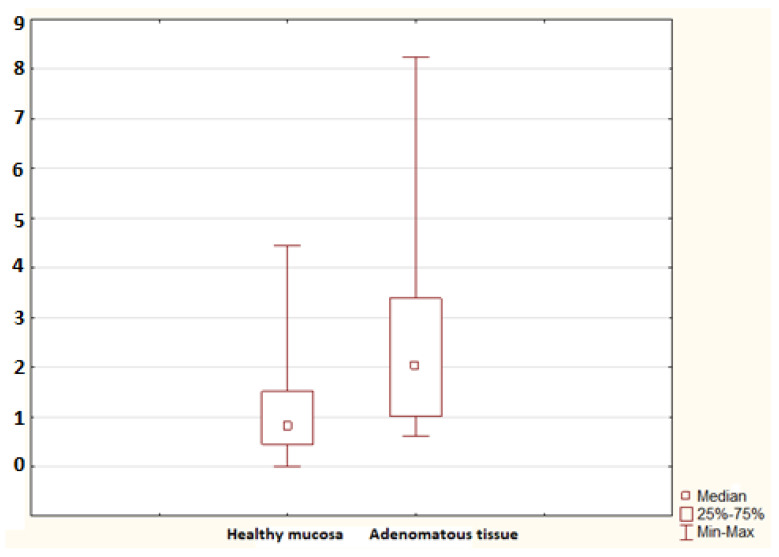
The mRNA for S100A11 in the healthy mucosa of the advanced adenoma group (healthy mucosa; 30 samples) and advanced adenomatous tissue (adenomatous tissue; 9 samples). A statistically significant difference was found (*p* = 0.029).

No statistically significant difference in the mRNA for S100A9 was found between controls and the non-advanced adenoma group (*p* = 0.052), nor was any difference identified between controls and the advanced adenoma group (*p* = 0.694). A significant difference in the mRNA for S100A11 was confirmed between controls and the advanced adenoma group (*p* = 0.005); no difference was found between controls and the non-advanced adenoma group (*p* = 0.110; Figure 2). No difference in the mRNA for S100P was found between controls and the non-advanced adenoma group and between controls and the advanced adenoma group (*p* = 0.987 and *p* = 0.558, respectively). The advanced adenoma group was analysed further. The mRNA for each S100 protein in the healthy mucosa of the advanced adenoma group was compared to the mRNA for each S100 protein in the tissue of advanced colorectal neoplasia. No difference was found in the mRNA for S100A4 and S100P (*p* = 0.113 and *p* = 0.138, respectively). Significant differences were identified in the mRNA for S100A6 (*p* = 0.002; Figure 3), S100A8 (*p* = 0.002; Figure 4), S100A9 (*p* = 0.021; Figure 5) and S100A11 (*p* = 0.029; Figure 6). 

Cycle threshold (Ct) values for the specific S100 protein in each tested group are included in Tables S1–S3 (Supplementary Material).

Three differences (out of 18 groups tested: in controls, the non-advanced adenoma group, the advanced adenoma group; six mRNAs for S100 proteins evaluated in each group) were confirmed between men and women in the mRNA for the following S100 proteins: S100A4 in the non-advanced adenoma group (normal distribution of data; mean ± standard deviation (SD) for men, 0.605 ± 0.289; women, 1.198 ± 0.709; *p* = 0.034), S100A9 in the advanced adenoma group (abnormal distribution of data; median inter-quartile range (IQR) for men, 0.332, 0.233; for women, 0.613, 0.913; *p* = 0.004) and S100A11 in the advanced adenoma group (normal distribution of data; mean ± SD for men, 0.665 ± 0.501; for women, 1.719 ± 1.195; *p* = 0.004).

## 3. Discussion

Our current study has brought a new priority insight into the mRNA dysregulation of S100 proteins in precancerous colorectal neoplasia based on the preliminary data. Patients with adenoma had a significantly higher mRNA expression for S100 proteins not only in the pathological adenomatous tissue but also in the healthy mucosa at different sites of the large bowel (with normal endoscopic appearance and normal histology). This is an important proof of the biological instability of all the intestinal mucosa in patients with premalignant colorectal conditions. This finding is in agreement with our recent study on the mucosal microbiome in colorectal neoplasia. Patients with previous and current colorectal neoplasia had a fully comparable bacteriocinogenic pattern [3]. 

Genes encoding specific S100 proteins are located on chromosomes 1q21 (S100A) [17] and 4p16 (S100P) [18]. Transcription of mRNA from the DNA sequence is regulated at several levels, yet only a few transcription factors have been recognised so far. Cis-regulatory elements are a highly enriched subset of the non-coding regions of the genome [19]. They play a critical role in the transcriptional activation of the S100P gene [20]. S100A protein genes are predominantly regulated at the epigenetic level. Hypomethylation of the S100A4 gene, accompanied by an increased protein level, was demonstrated in colon cancer [21]. DNA hypomethylation also contributes to the regulation of the expression of S100A6, S100A8 and S100A11 in various cancers [22,23,24]. 

Surprisingly, we found no significant difference in the tissue mRNA for S100A4 between controls and both non-advanced and advanced adenoma groups. This can be explained by its importance being marginal in the tumor biology of adenomas as precancerous conditions. The mRNA for S100A4 and S100A4 proteins has a prognostic significance in colorectal cancer [15,25]. S100A4 (metastatin-1, calvasculin) is a multifunctional protein localized in the nucleus, cytoplasm and extracellular space. It is strongly associated with metastatic tumor progression [10,26]. S100A4, when released into the extracellular space, enables angiogenesis through stimulation of endothelial cell motility and through activation of matrix metalloproteinase expression, which cleaves proteins of the extracellular matrix and thereby facilitates cell invasion into adjacent tissues [26]. Experimental specific knockdown of S100A4 strongly suppresses cell growth, migration and invasion activities in colorectal cell lines. Therefore, inhibition of S100A4 might be a novel approach to the treatment of colorectal cancer [27].

The S100A6 protein, also known as calcyclin, is expressed as a 90-amino-acid protein in humans [28,29]. In relationship to cancer, the S100A6 protein co-regulates the proliferation and apoptosis of cells [29,30,31]. Importantly, p53, as a genome guardian, suppresses the S100A6 gene. Therefore, insufficient suppression of S100A6 (e.g., by p53 mutants derived from human cancers) leads to the overexpression of S100A6 and subsequently contributes to the loss of cell cycle control [32]. Komatsu et al. described the overexpression of S100A6 in surgically resected human colorectal adenocarcinomas. They used Western blot analysis and immunohistochemical analysis [33]. Our study focused on the assessment of the gene expression of S100A6 and confirmed that the mRNA for S100A6 increased significantly in the healthy mucosa of those who had an advanced adenoma in their colon or rectum at the time of the study compared to controls. Our study also showed that there was a further significantly increased expression of S100A6 in the tissue of advanced colorectal adenoma patients compared to the normal healthy mucosa of those with an advanced adenoma. 

S100A8 (calgranulin A) is a calcium- and zinc-binding protein that plays a principal role in the regulation of an inflammatory and immune response. S100A8 is a potent amplifier of cancer development and tumor spread [34,35]. The S100A9 protein (calgranulin B) has a strong proinflammatory impact, stimulates chemotaxis, exerts a tumor-promoting effect in colorectal carcinoma and stimulates its neoplastic progression [36]. In our current study, we found a statistically significant difference in the tissue mRNA for S100A8 but no significant difference in S100A9 between controls and patients with adenomas. Previously, we found increased serum S100A8 but not S100A9 in colorectal neoplasia patients compared to controls [11]. We can hypothesize that S100A8 plays a prominent regulatory role in tumor biology in comparison with S100A9, yet a larger study could identify more relationships and differences in S100A9 as well. 

Calprotectin is a heterocomplex of S100A8/S100A9. Heterodimerization with S100A8 stabilizes S100A9 [8,9,10]. We did not investigate calprotectin in our current study. However, its role in colorectal cancer is well-known. The heterocomplex of S100A8/S100A9 may induce tumorous invasion. The effect of calprotectin on tumor cells is dependent on the concentration: at high concentrations, the heterocomplex of S100A8/S100A9 exerts an apoptotic effect [37], but at low concentrations, calprotectin promotes tumor cell growth [38]. Increased tissue expression of calprotectin is an early step in the neoplastic transformation during colorectal carcinogenesis. Its expression is closely related to an inflammatory response and indicates a biological link between inflammation and neoplastic transformation in colorectal cancer [39]. In clinical practice, calprotectin was suggested as a biomarker of colorectal cancer [40]. Fecal calprotectin has a high negative predictive value for colorectal cancer and advanced adenomas of the large bowel [41]. 

S100A11, also known as S100C or calgizzarin, has been reported to contribute to inflammatory processes [42] and multiple neoplastic events in different types of cancers, including papillary thyroid cancer [43], ovarian cancer [44], cholangiocarcinoma [45], pancreatic carcinoma [46,47] and gastric cancer [48]. Takamatsu et al. concluded that extracellular S100A11 contributes significantly to the spread of the fibroblast population in pancreatic cancer, thus becoming a potential therapeutic target [49]. Koh et al. confirmed that S100A11 is upregulated by the hepatocyte growth factor through the NF-κB pathway in gastric cancer and plays a key role in the cell proliferation and further invasion of gastric carcinoma [48]. Calgizzarin was also studied in colorectal neoplasia: Melle et al. used peptide fingerprint mapping and immunohistochemistry: they confirmed a different expression of S100A11 in the normal colonic epithelium, adenoma and colorectal carcinoma [50]. Our study confirmed significantly increased mRNA for S100A11 in the normal intestinal mucosa, which once again shows the instability of the mucosa in those with colorectal neoplasia. A further significant increase in the mRNA for S100A11 was observed in the tissue of an advanced adenoma when compared to the healthy mucosa in those with an advanced adenomatous polyps.

S100 calcium-binding protein P (S100P) is involved in the regulation of the cell cycle and differentiation. S100P stimulates growth and promotes the invasion and metastasis of colorectal cancer [51,52,53]. The S100P protein has been suggested as a potential novel prognostic biomarker of colorectal cancer [54]. Upregulation of S100P was described in colorectal adenoma compared to normal tissue [55]. We were not able to support this finding as we found no difference in the tissue mRNA for S100P between controls, non-advanced adenoma patients and advanced adenoma patients. 

Differences in the mRNA for S100A4 in the non-advanced adenoma group and the mRNA for S100A9 and S100A11 in the advanced adenoma group between men and women were confirmed. The results are not convincing, and no firm conclusions can be drawn based on this; however, these data does not support the hypothesis that S100 proteins could contribute to the known higher incidence of colorectal neoplasia in men. 

We are aware of the possible limitations of our pilot study. The small number of individuals enrolled in the study (6 controls, 6 patients with a non-advanced adenoma and 10 subjects with an advanced adenoma) is the major limitation. Yet, three biopsies in each individual were taken (caecum, transverse colon, rectum), and a fourth biopsy was obtained in 18/39 mRNA assessments; therefore, the final numbers were sufficient for statistical analysis. 

## 4. Methods

### 4.1. Subjects

A total of 22 individuals referred for a colonoscopy were included in the study. The group consisted of 6 controls (healthy subjects with normal findings on colonoscopy: 4 men and 2 women; mean age 52 ± 12 years), 6 patients with a non-advanced adenoma (3 men and 3 women; mean age 55 ± 7 years) and 10 patients with an advanced adenoma (5 men and 5 women; mean age 69 ± 9 years). Advanced adenomas are defined as adenomas containing villous component and/or being at least 10 mm in size and/or containing high-grade dysplasia [56]. Inclusion criteria were: population at an average risk for colorectal carcinoma, negative history of inflammatory bowel disease and/or colorectal neoplasia and absence of antibiotic and/or probiotic treatment within the last four weeks before the study.

### 4.2. Endoscopic Procedure

All patients received an oral cleansing agent: 20/22 individuals (91%) were prepared with polyethyleneglycol. Two healthy subjects were prepared with natrium picosulfate. Mucosal biopsies were obtained during the diagnostic and/or therapeutic colonoscopy from the normal mucosa in the caecum, transverse colon and rectum. Additional targeted samples were taken from the advanced adenomatous tissue in 9/10 patients (90%). Single-use radial jaw 4 biopsy forceps (Boston Scientific, Natick, MA, USA) were used for biopsies. Biopsy specimens were put in separate vials with RNAprotect Tissue Reagent (QIAGEN, Hilde, Germany) immediately.

### 4.3. RNA Isolation qPCR

The biopsy specimens were homogenized using the Precellys 24 homogenizer (Bertin Instruments, Bretonneux, France). Total cellular RNA was extracted using the TRIzol reagent (Invitrogen, Carlsbad, CA, USA) according to the method described by Chomczynski and Sacchi [57]. RNA was reverse-transcribed using a cDNA reverse transcription kit [57]. Gene expression was evaluated with TaqMan Gene Expression Assays (POLR2A Hs00172187_m1, S100A4 Hs00243202_m1, S100A6 Hs00170953_m1, S100A8 Hs00374263_m1, S100A9 Hs00610058_m1, S100A11 Hs00271612_m1 and S100P HS00195584_m1). Gene expression was analyzed using the QuantStudio 6 real-time PCR system (all obtained from Applied Biosystems, Foster City, CA, USA) [58]. Results were normalized to POLR2A RNA expression. mRNA levels were calculated using the comparative Ct method (ΔΔCt method) [58]. The comparative Ct method uses three sequential mathematical operations: 1st step, ΔCt = Ct_(target gene)_ – Ct_(reference gene)_; 2nd step, ΔΔCT = ΔCT_(target sample)_ – ΔCT_(reference sample)_; and 3rd step, Q = 2^-ΔΔCt [59].

### 4.4. Statistical Analysis

Data were treated statistically by means of descriptive statistics. Data with an abnormal distribution (majority) were analyzed by the non-parametric Mann–Whitney test, and data with a normal distribution were analyzed by a parametric unpaired *t*-test. Statistica software (version 13, 2013, Tulsa, OK, USA) was used. ANOVA was used as well (one-way and two-way ANOVA, Kruskal–Wallis one-way ANOVA on ranks, all pairwise multiple-comparison procedures with Dunn’s method and the Holm–Sidak method) using SigmaStat software (version 3.1, Jandel Corp, Erkrath, Germany).

### 4.5. Ethical Issues

The project was conducted according to the Declaration of Helsinki and was approved by the Joint Ethics Committee (Charles University, Faculty of Medicine in Hradec Kralove & University Hospital Hradec Kralove) under the number 202006 S20P). For all data obtained, all personal identification information was deleted in compliance with the Czech laws for protection of confidentiality. All patients included in the study were provided with adequate information and signed informed consent. 

## 5. Conclusions

The mRNA dysregulation of different S100 proteins in colorectal adenomas has been confirmed. Abnormal mRNA expression of the S100 proteins studied was observed not only in the pathological tissue but also in the morphologically normal mucosa of the large intestine.

## Data Availability

Not applicable.

## Supplementary Materials

The following are available online. Tables S1: Cycle treshold (Ct) values for each S100 protein in controls; Table S2: Cycle treshold (Ct) values for each S100 protein in non-advanced (non-adv) adenoma group; Table S3: Cycle treshold (Ct) values for each S100 protein in advanced adenoma group.

## Sample Availability

Samples of the compounds are not available from the authors.
