# Supplementary material for: Tissue mRNA for S100A4, S100A6, S100A8, S100A9, S100A11 and S100P Proteins in Colorectal Neoplasia: A Pilot Study
Source: Molecules. 2021 Jan 14;26(2):402. doi: 10.3390/molecules26020402 (PMC7828666; doi:10.3390/molecules26020402)
Supplement: Supplementary file 1 [file molecules-26-00402-s001.pdf]

Table S1. Cycle treshold (Ct) values for each S100 protein in controls.

Control number	Localization	Ct POLR2A	Ct S100A4	Ct S100A6	Ct S100A8	Ct S100A9	Ct S100A11	Ct S100P
1	C	24.789	25.414	18.999	28.588	27.795	23.359	27.991
	T	24.525	24.826	19.652	30.292	28.314	24.228	26.749
	R	24.888	24.344	19.986	30.884	28.888	23.457	24.333
2	C	25.343	26.133	19.921	32.665	30.550	24.580	27.672
	T	26.070	27.221	21.762	33.616	31.074	27.013	29.227
	R	25.281	25.041	20.337	31.488	29.428	24.535	25.438
3	C	25.705	26.849	21.140	30.480	29.164	25.327	28.093
	T	25.520	26.324	20.677	30.056	28.546	25.135	26.307
	R	25.956	26.671	21.231	31.674	29.135	25.264	25.650
4	C	25.568	25.952	19.371	28.679	27.564	23.555	28.105
	T	26.737	28.933	23.153	31.792	31.042	29.051	30.000
	R	26.804	27.526	21.771	32.098	30.808	26.527	27.293
5	C	25.600	26.980	21.106	29.907	29.124	25.824	28.461
	T	26.422	27.962	22.167	31.274	30.537	27.499	28.967
	R	25.983	27.848	21.826	32.477	31.325	26.568	26.021
6	C	24.981	28.852	22.783	31.022	30.511	28.968	27.450
	T	26.652	28.373	23.103	32.974	31.539	28.268	30.0766
	R	26.647	27.987	23.014	33.548	32.008	27.516	26.851

POLR2A: gene encoding RNA polymerase II, the polymerase responsible for synthesizing mRNA. C: caecum, T: transverse colon, R: rectum

Table S2: Cycle treshold (Ct) values for each S100 protein in non-advanced (non-adv) adenoma group

Non-adv adenoma number	Localization	Ct POLR2A	Ct S100A4	Ct S100A6	Ct S100A8	Ct S100A9	Ct S100A11	Ct S100P
1	C	25.248	25.961	20.196	29.657	28.036	24.654	26.459
	T	24.869	24.891	19.782	30.597	29.087	23.528	26.774
	R	25.029	25.235	20.022	30.134	29.310	23.380	24.436
2	C	24.978	25.253	19.538	28.178	27.274	23.399	27.616
	T	26.294	27.053	22.527	32.847	30.382	26.793	30.563
	R	24.540	23.836	19.360	28.803	28.264	22.688	25.141
3	C	26.839	27.668	21.942	29.089	29.304	24.212	29.672
	T	25.551	28.581	23.218	31.925	30.123	27.895	22.735
	R	26.448	26.995	20.845	30.684	29.805	23.926	25.802
4	C	24.612	27.133	20.781	29.152	28.289	26.820	28.583
	T	25.641	26.852	21.515	29.797	29.086	25.579	29.252
	R	24.288	25.675	20.284	30.235	28.502	24.486	25.068
5	C	25.439	27.626	20.906	31.026	29.070	28.394	29.220
	T	25.489	26.566	19.467	28.515	28.169	23.837	27.688
	R	25.190	26.255	19.192	29.457	28.890	23.837	24.956
6	C	25.801	26.597	20.122	27.616	27.908	24.997	28.397
	T	25.663	26.923	20.704	29.324	29.245	25.992	29.499
	R	25.902	25.768	20.533	29.427	28.964	24.112	25.630

POLR2A: gene encoding RNA polymerase II, the polymerase responsible for synthesizing mRNA. C: caecum, T: transverse colon, R: rectum

Table S3: Cycle treshold (Ct) values for each S100 protein in advanced adenoma group.

Advanced adenoma number	Localization	Ct POLR2A	Ct S100A4	Ct S100A6	Ct S100A8	Ct S100A9	Ct S100A11	Ct S100P
1	C	24.975	25.888	19.801	29.912	28.335	23.982	27.121
	T	24.195	25.015	18.752	28.212	27.435	22.332	22.861
	R	25.095	26.469	20.746	32.217	30.286	25.085	24.865
2	C	24.627	25.280	18.638	26.869	26.426	22.601	25.212
	T	25.928	30.640	24.754	32.446	31.784	34.551	26.927
	R	25.056	25.623	19.563	31.421	30.200	24.053	23.605
3	C	25.666	25.524	20.563	30.612	30.146	24.456	28.511
	T	24.843	24.486	19.056	29.988	29.441	23.253	27.587
	R	24.779	24.362	18.966	31.383	29.846	22.807	25.319
4	C	25.322	25.863	19.774	28.984	28.325	23.738	27.069
	T	25.309	25.895	20.000	30.834	29.440	24.357	27.902
	R	25.494	26.111	20.498	32.015	30.844	24.829	28.871
5	C	25.770	26.403	20.668	30.420	29.078	24.801	28.769
	T	26.160	26.880	21.496	31.271	29.968	26.106	29.020
	R	25.407	25.736	20.019	31.667	29.467	24.532	25.426
6	C	25.107	25.552	19.336	28.139	27.459	22.195	27.076
	T	26.001	26.832	20.517	30.349	29.712	23.906	27.101
	R	25.432	25.702	20.095	31.975	29.375	23.226	23.955
7	C	25.679	25.894	19.609	27.474	26.259	22.096	31.227
	T	25.430	27.961	20.043	28.946	27.955	22.207	27.952
	R	25.564	25.103	20.871	33.166	29.909	22.680	26.171
8	C	26.782	27.798	21.395	31.094	29.361	24.969	28.267
	T	26.639	27.461	20.764	32.270	26.960	23.995	27.454
	R	26.388	26.742	21.352	32.278	30.141	24.690	26.038
9	C	25.869	27.621	21.256	29.600	30.080	26.764	28.601
	T	25.745	26.778	21.307	29.697	29.937	25.867	28.250
	R	25.203	26.133	19.838	29.799	29.911	24.697	24.651
10	C	24.620	26.310	19.701	29.290	28.767	25.822	28.738
	T	24.944	28.943	21.960	27.667	28.279	21.667	26.617
	R	24.610	26.113	20.326	30.24	29.870	26.065	24.284

POLR2A: gene encoding RNA polymerase II, the polymerase responsible for synthesizing mRNA. C: caecum, T: transverse colon, R: rectum
